# Higher scanning frequency is correlated with less fear of hypoglycemia in type 1 diabetes patients using isCGM

**DOI:** 10.3389/fendo.2022.996933

**Published:** 2022-10-06

**Authors:** Jerzy Hohendorff, Przemyslaw Witek, Michal Kania, Maria Sudol, Katarzyna Hajduk, Adam Stepien, Katarzyna Cyganek, Beata Kiec-Wilk, Tomasz Klupa, Maciej T. Malecki

**Affiliations:** ^1^ Department of Metabolic Diseases, Jagiellonian University Medical College, Krakow, Poland; ^2^ Department of Metabolic Diseases and Diabetology, University Hospital in Krakow, Krakow, Poland; ^3^ Unit of Rare Metabolic Diseases, Department of Metabolic Diseases and Diabetology, University Hospital in Krakow, Krakow, Poland

**Keywords:** continous glucose monitoring, intermittently scanned CGM, ambulatory glucose profile (AGP), fear of hypoglycemia, type 1 diabetes, time in range, time below range

## Abstract

**Background:**

Frequent scanning of intermittently scanned continuous glucose monitoring (isCGM) devices is associated with improvements in glycemic indices. Limited data is available for its correlation with fear of hypoglycemia (FOH), an established factor affecting quality of life and glycemic control in type 1 diabetes (T1DM).

**Aim:**

The aim of the study was to analyze the association of sensor scanning frequency with FOH and glycemic indices in T1DM patients using isCGM.

**Subjects and methods:**

T1DM patients using isCGM were eligible. Clinical data and Ambulatory Glucose Profile (AGP) reports were obtained from medical records. At outpatient visits, AGP of last 14 days prior to visit were analyzed and FOH was assessed using Hypoglycemia Fear Survey II (HFS II).

**Results:**

We included 77 consecutive T1DM patients (58 females, 19 males). Mean age was 34.1 ± 10.2 years and mean T1DM duration was 14.7 ± 12.0 years. Baseline mean glycemic indices were as follows: mean glucose - 155.8 ± 29.8 mg/dL; GMI - 53.3 ± 7.5 mmol/mol; TIR - 66.4 ± 17.8%; TBR70 - 4.5 ± 4.1%; TBR54 - 0.6 ± 1.2%; TAR180 - 29.2 ± 17.9%; TAR250 - 9.6 ± 10.4%; %CV - 36.7 ± 8.3. Average scanning frequency was 13.8 ± 7.8 scans/day. Mean HFS II scores were 16.1 ± 7.2 and 18.7 ± 12.2 in behavior and worry subscale, respectively. Correlation was confirmed between scanning frequency and mean glucose, GMI, TIR, TBR70, TAR180, TAR250, %CV and HFS II total, and HFS II - B (p<0.05 for all statistics).

**Conclusions:**

For the first time, we report that higher scanning frequency is associated not only with better glycemic indices but also with less FOH in T1DM adult patients using isCGM.

## Introduction

Globally, more than 530 million people are living with diabetes, including approximately 9,000,000 (2%) diagnosed with type 1 diabetes (T1DM) (1, 2). In people with T1DM, a strong association is evident between frequent self-monitoring of blood glucose (SMBG) and glycemic control as assessed by glycated hemoglobin A1c (HbA1c) (3, 4). Patients with diabetes on intensive insulin therapy (IIT) with MDI or insulin pumps are advised to perform at least 4 SMBG tests per day or use continuous glucose monitoring (CGM) devices – either intermittently-scanned CGM (isCGM) or real-time CGM (rtCGM) (5, 6). The only isCGM currently available are the FreeStyle Libre^®^ system and FreeStyle Libre 2 system (Abbott Diabetes Care Inc., USA) (7). Many published studies have reported on the frequency of daily scans and glycemic indices in patients using isCGM. Based on de-identified data it was shown within different populations that patients who perform more scans per day have lower mean glucose, lower glucose management index (GMI), spend more time in range (TIR) and less time above range (TAR) and time below range (TBR), as defined by the International Consensus on Time in Range and as visualized in ambulatory glucose profile (AGP) reports (8–12). Moreover, using isCGM is associated with less hospitalizations due to severe hypoglycemia or diabetic ketoacidosis (DKA), less workplace absenteeism, and higher quality of life (13–15). However, only limited data is available that examines the correlation of scanning frequency with fear of hypoglycemia (FOH), an established factor affecting quality of life and glycemic control in people with T1DM. Such an association has been reported in children and adolescents, but not in adults (16–18). As well as affecting glycemic control, FOH has been shown to be associated with high calorie intake and reduced physical activity (19). In this observational cohort study, our aim was to analyze the association between scanning frequency and FOH, as well as glycemic indices in T1DM patients using isCGM.

## Materials and methods

### Patients

T1DM patients, active isCGM users were recruited between October and December 2021 in a single outpatient academic clinic that provides diabetes care to patients in the University Hospital in Krakow, Poland. Data, such as age, sex, diabetes duration, type of therapy and presence of diabetic complications were obtained from medical records. As in Poland isCGM is reimbursted for T1DM patients aged ≤18 years only, thus all adult patients using isCGM cover all cost of sensors themselves. Women planning pregnancy or being pregnant were not involved in the study. The study was performed in accordance with the Declaration of Helsinki and was approved by local Bioethics Committee. All participants provided informed consent.

### Ambulatory glucose profile and scanning details

The FreeStyle Libre sensor measures interstitial glucose levels for up to 14 days (7). Data collected by sensors are uploaded by patients using the LibreLink smartphone app to the LibreView platform (Abbott Diabetes Care Inc., USA), which generates personal AGP reports. Glucose ranges as assessed were defined as: TIR 70-180 mg/dL (3.9-10.0 mmol/L), TBR70 <70 mg/dL (<3.9 mmol/L), and TAR180 >180 mg/dL (>10.0 mmol/L), in accordance with the international consensus ranges (12). Time spent in very high glucose and very low ranges defined as TAR250 >250 mg/dL (13.9 mmol/L) and TBR54 <54 mg/dL (<3.0 mmol/L) were assessed as well (12). Data on scanning frequency was obtained from patients’ personal reports generated in LibreView. Last 14 days were analyzed prior to a visit in outpatient clinic. Data was included to analyses only if percentage of time CGM was active was at least 70%.

### Fear of hypoglycemia

At the study visit, FOH was assessed using Hypoglycemia Fear Survey II (HFS II), which is a validated measure of FOH in adults with T1DM. HFS II contains both a worry subscale (HFS II – W) and a separate behavior subscale (HFS II – B) (20).

### Statistical analysis

Statistical analysis was performed using Statistica, version 13, TIBCO Software Inc., CA, USA. Basic descriptive statistics were calculated for the entire study group, patients treated with MDI and insulin pump users, and for five scan-rate groups, each containing 20% of subjects from least to most scanners. Parametric t test or nonparametric U test were performed, where applicable, to describe clinical characteristics and differences between patients on MDI and pump users, while for nominal variables the Fisher’s exact test was used. Correlations were analyzed between scanning frequency, glycemic control indices and FOH. Moreover, multiple regression model was built to find factors that affect HFS. A p<0.05 was considered to be significant.

## Results

### Characteristics of the study group

77 (58 female, 19 male) adults with T1DM were included in the study. Of these, 39 were treated with MDI, and 38 were insulin pump users. The mean age of subjects was 34.1 ± 10.2 years and mean T1DM duration was 14.7 ± 12.0 years. In the study group, there were 3 patients with a history of episode of severe hypoglycemia and 5 with history of DKA in the previous 12 months. There were no patients with diagnosed advanced chronic complications. Detailed characteristics of the study group are shown in **Table 1**.

**Table 1:** Clinical characteristics of the study group.

	Entire group	CSII	MDI	p
Number of cases, n	77	38	39	N/A
Sex female/male, n	58/19	34/4	24/15	<0.01
Age, years	34.1 ± 10.2	33.2 ± 8.9	35.1 ± 11.3	0.42
Diabetes duration, years	14.7 ± 12.0	17.2 ± 11.0	12.3 ± 12.5	0.07
BMI, kg/m2	23.7 ± 3.2	23.6 ± 3.5	23.9 ± 2.9	0.69
Mean glucose, mg/dL	155.8 ± 29.8	156.5 ± 26.9	155.2 ± 32.8	0.86
GMI, %	7.03 ± 0.68	7.05 ± 0.63	7.01 ± 0.73	0.81
GMI, mmol/mol	53.3 ± 7.5	53.6 ± 6.9	53.1 ± 8.2	0.78
CV, %	36.7 ± 8.3	38.0 ± 8.2	35.3 ± 8.2	0.15
TAR250, %	9.6 ± 10.4	10.0 ± 10.1	9.2 ± 10.7	0.74
TAR180, %	29.2 ± 9.7	29.4 ± 16.1	28.9 ± 19.7	0.90
TIR, %	66.4 ± 17.8	65.3 ± 16.2	67.5 ± 19.4	0.60
TBR70, %	4.5 ± 4.1	5.3 ± 4.4	3.8 ± 3.6	0.11
TBR54, %	0.6 ± 1.2	0.7 ± 1.1	0.5 ± 1.2	0.59
Scanning frequency, n/d	13.8 ± 7.8	14.0 ± 8.2	13.6 ± 7.5	0.82
HFS II	34.7 ± 16.8	36.3 ± 16.7	33.2 ± 16.9	0.42
HFS II – B	16.1 ± 7.2	15.5 ± 6.2	16.6 ± 8.0	0.49
HFS II – W	18.7 ± 12.2	20.8 ± 13.4	16.6 ± 10.7	0.13

Data shown as n – number of cases or mean ± SD. BMI, Body mass index; CV, Coefficient of variation; GMI, Glucose management indicator; HFS II, Hypoglycemia Fear Survey II; HFS II – B, HFS II Behavior subscale; HFS II – W, HFS II Worry subscale; TIR, Time in range 70-180 mg/dL; TAR250, Time above range >250 mg/dL; TAR180, Time above range >180 mg/dL; TBR70, Time below range <70 mg/dL; TBR54; Time below range <54 mg/dL.

### Glycemic indices

**Figure 1:**
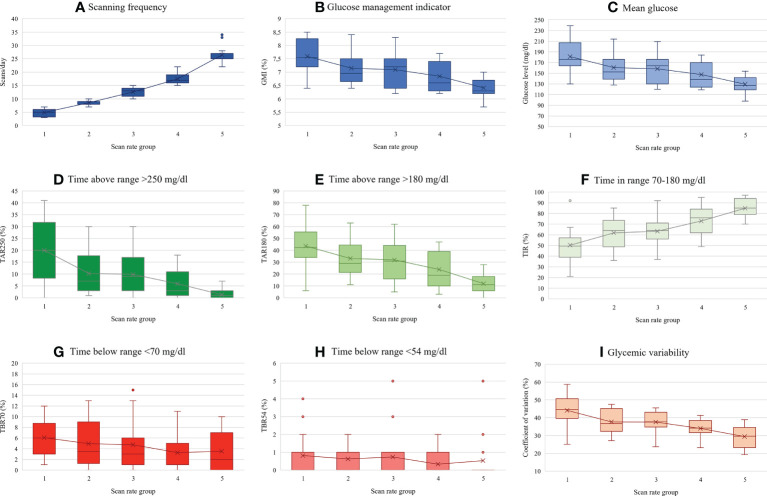
Glycemic indices across scan rate groups (each group represents 20% of subjects (first 2 groups – n=16, next 3 groups – n=15). **(A)** Scanning frequency, **(B)** Glucose management indicator, **(C)** Mean glucose, **(D)** Time above range >250 mg/dl, **(E)** Time above range >180 mg/dl, **(F)** Time in range 70-180 mg/dl, **(G)** Time below range <70 mg/dl, **(H)** Time below range <54 mg/dl, **(I)** Glycemic variability expressed as coefficient of variation. Data shown as mean and interquartile range.

**Figure 2:**
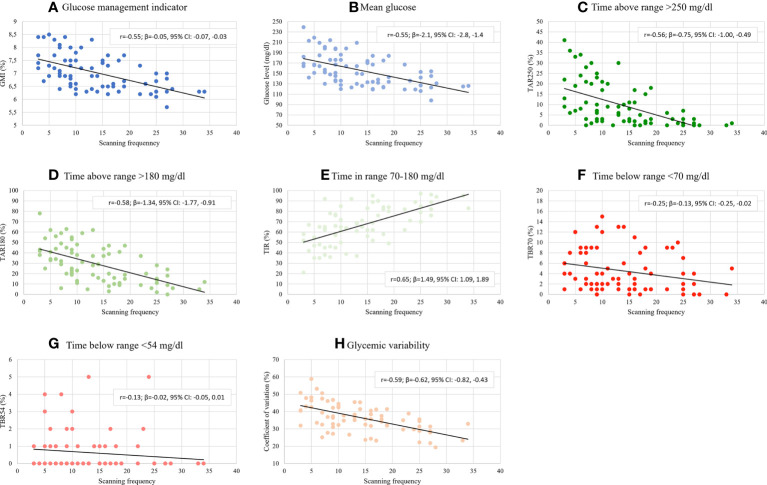
Glycemic indices by scanning frequency. **(A)** Glucose management indicator, **(B)** Mean glucose, **(C)** Time above range >250 mg/dl, **(D)** Time above range >180 mg/dl, **(E)** Time in range 70-180 mg/dl, **(F)** Time below range <70 mg/dl, **(G)** Time below range <54 mg/dl, **(H)** Glycemic variability expressed as coefficient of variation. β - beta coefficient, r - Pearson correlation coefficient.

The study participants performed on average 13.8 ± 7.8 scans/day, median 13 scans/day. Mean glucose was 155.8 ± 29.8 mg/dL and GMI 7.03 ± 0.68% (53.3 ± 7.5 mmol/mol). Mean TIR was 66.4 ± 17.8%, TBR70 was 4.5 ± 4.1%, TBR54 was 0.6 ± 1.2%, TAR180 was 29.2 ± 17.9%, and TAR250 was 9.6 ± 10.4%. Mean glycemic variability expressed as coefficient of variation (CV) was 36.7 ± 8.3%. Detailed data on glycemic indices across the five scan-rate groups is shown in **Table 2** and in **Figure 1**. As expected, significant correlations were found between scanning frequency and mean glucose (r=-0.54, β=-2.1, 95% CI: -2.8, -1.4), GMI (r=-0.55, β=-0.05, 95% CI: -0.07, -0.03), TIR (r=0.65, β=1.49, 95% CI: 1.09, 1.89), TBR70 (r=-0.25, β=-0.13, 95% CI: -0.25, -0.02), TAR180 (r=-0.58, β=-1.34, 95% CI: -1.77, -0.91), TAR250 (r=-0.56, β=-0.75, 95% CI: -1.00, -0.49), and %CV (r=-0.59, β=-0.62, 95% CI: -0.82, -0.43). No significant correlation was evident between the scanning rate and TBR54 (r=-0.13, β=-0.02, 95% CI: -0.05, 0.01) (**Figure 2**).

**Table 2:** Glycemic indices and HFS II according to the scan rate group.

Scan rate group	Scanning frequency	Mean glucose	GMI		Glucose CV	TBR54	TBR70	TIR	TAR180	TAR250	HFS II	HFS II - B	HFS II - W
	(scans/day)	(mg/dL)	(mmol/mol)	(%)	(%)	(%)	(%)	(%)	(%)	(%)			
Group 1	4.9 ± 1.5	181.2 ± 30.0	59.6 ± 7.0	7.6 ± 0.6	44.1 ± 8.8	0.8 ± 1.2	6.1 ± 3.8	50.3 ± 16.2	43.6 ± 16.6	20.0 ± 12.2	42.5 ± 19.1	20.0 ± 7.7	22.5 ± 14.4
Group 2	8.6 ± 1.0	160.6 ± 25.3	54.5 ± 6.7	7.2 ± 0.6	37.7 ± 6.9	0.6 ± 1.1	4.9 ± 4.4	61.9 ± 13.7	33.2 ± 15.0	10.3 ± 9.3	34.3 ± 19.3	13.8 ± 7.7	20.5 ± 15.2
Group 3	12.6 ± 1.8	158.3 ± 28.2	54.2 ± 7.2	7.1 ± 0.7	37.6 ± 6.5	0.7 ± 1.4	4.7 ± 4.9	63.3 ± 14.5	31.9 ± 16.9	9.9 ± 8.6	32.8 ± 15.1	16.7 ± 7.3	16.1 ± 9.4
Group 4	17.5 ± 1.8	147.6 ± 24.1	51.2 ± 6.3	6.8 ± 0.6	34.0 ± 5.5	0.3 ± 0.6	3.3 ± 3.5	72.9 ± 14.7	23.9 ± 15.5	5.9 ± 6.4	33.3 ± 14.2	15.3 ± 6.6	18.0 ± 9.3
Group 5	26.5 ± 3.3	129.4 ± 14.8	46.7 ± 4.0	6.4 ± 0.5	29.4 ± 5.7	0.5 ± 1.4	3.5 ± 3.7	84.9 ± 8.6	11.9 ± 8.0	1.3 ± 2.0	30.3 ± 14.4	14.5 ± 5.2	15.9 ± 11.4

Scan groups consists of n=16 (first 2 groups) and n=15 (next 3 groups). All data are shown as mean ± SD. CV, Coefficient of variation; GMI, Glucose management indicator; HFS II, Hypoglycemia Fear Survey II; HFS II – B, HFS II Behavior subscale; HFS II – W, HFS II Worry subscale; TIR, Time in range 70-180 mg/dL; TAR250, Time above range >250 mg/dL; TAR180, Time above range >180 mg/dL; TBR70, Time below range <70 mg/dL; TBR54; Time below range <54 mg/dL.

### Fear of hypoglycemia

**Figure 3:**
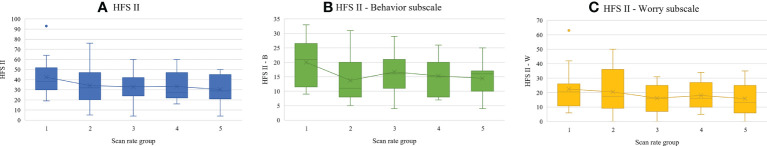
Fear of hypoglycemia across scan rate groups (each group represents 20% of subjects (first 2 groups – n=16, next 3 groups – n=15). **(A)** HFS II, **(B)** HFS II - Behavior subscale, **(C)** HFS II - Worry subscale. Data shown as mean and interquartile range.

**Figure 4:**
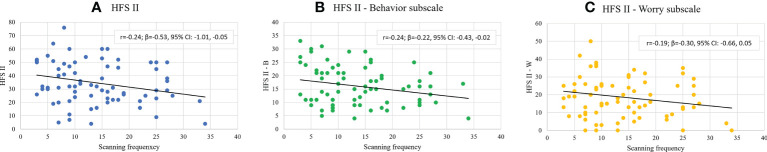
Fear of hypoglycemia by scanning frequency. **(A)** HFS II, **(B)** HFS II - Behavior subscale, **(C)** HFS II - Worry subscale. β - beta coefficient, r - Pearson correlation coefficient.

The mean total HFS II score was 34.7 ± 16.6, with 16.1 ± 7.2 and 18.7 ± 12.2 scores for the behavior and worry subscales, respectively (**Table 1** and **Figure 3**). Data on FOH across the five scan-rate groups is shown in **Table 2**. Significant correlations were found between scanning frequency and overall HFS II score (r=-0.25, β=-0.53, 95% CI: -1.01, -0.05), and with the HFS II–B subscale (r=-0.24, β=-0.22, 95% CI: -0.43, -0.02). No significant correlation was found with the HFS II–W subscale (r=-0.19, β=-0.30, 95% CI: -0.66, 0.05) (**Figure 4**). In multiple regression analyzes, no significant association was observed between HFS II scores with: gender, or type of insulin therapy (MDI or insulin pumps).

## Discussion

In this single center observational cohort study, we have examined association between scanning frequency and FOH, and glycemic indices in adults with T1DM treated with insulin pumps or MDI. For the first time, we have found that scanning frequency is negatively correlated with FOH in adults with T1DM. We have shown that increased daily scan rates are associated with reduced fear of hypoglycemia for people with T1DM, as assessed by HFS II scores. Significant negative correlations were found in terms of HFS II total scores and the behavior subscale. No correlation between scanning frequency and worry subscale was demonstrated, although the observed scores were lower at the highest scan rate.

The first randomized clinical study to evaluate clinical effectiveness of isCGM was the IMPACT trial (13). In that study, using isCGM was associated with significant improvement in glycemic outcomes, particularly reduction in time spent in hypoglycemia, and improvement in treatment satisfaction score, but HFS II scores did not differ between intervention and control group (13). Such findings were confirmed in the FUTURE study, in which the impact of isCGM on quality of life (QoL) was assessed in real-world conditions, and showed that, after initiation of isCGM, treatment satisfaction increased, while QoL was maintained (15). Moreover, after initiation of isCGM, hospitalizations due to hypoglycemia and/or DKA were reduced, and less workplace absenteeism was observed (15). Authors of the FUTURE study concluded that FOH and treatment satisfaction were not different subgroups with different scan frequencies (no detailed results were provided) (15).

To the best of our knowledge, there is no published data on the association between scanning frequency and FOH in adults. In children and adolescents (aged 13-19 years) the frequency of isCGM use was negatively correlated with worry and positively with behavior assessed by the Hypoglycemia Fear Survey – Child version tool (16). FOH is an important factor influencing QoL and glycemic control, thus, any strategy that could lower FOH is potentially of clinical value (17, 18). In the STAR 3 randomized trail it was shown that sensor-augmented pump therapy (SAPT) when compared with MDI+SMBG had significant advantages for reducing FOH (21). In another clinical study on SAPT, FOH scores tended to be lower for SAPT users, but results were statistically insignificant (22).

Our study also confirms previous findings on the association between scanning frequency and glycemic indices (8–11). Most previous real-world studies on scanning frequency were based on de-identified data stored in the cloud, thus no clinical characteristics of study subjects could be examined. Our well-characterized study group consisted of adult patients with T1DM, half of them treated with insulin pumps. In that group, a significant imbalance in terms of gender could be seen (**Table 1**). However, in additional analyses, gender and type of insulin therapy were not found as significant factors affecting FOH. The mean scan rate in our group was above 13 scans per day, which is comparable with the international data (8). However, the number of daily scans performed by the wider international group was lower than observed within the larger Polish cohort, as reported previously by us based on de-identified data (>21 scans per day) (8). Nevertheless, a mean GMI of 7.03% is almost identical to earlier reported eA1c for the same previously reported national cohort (7.04%) and lower than observed in several other countries (7.49%) (8). This data could suggest the influence of country-specific factors on the observed results. First, in Poland, the great majority of subjects using CGM devices are people with T1DM. Second, because in Poland isCGM is partially reimbursed only for people with T1DM under the age of 18 years and not for adults, one could hypothesize that in the adult population it is preferentially used by patients with higher socioeconomic status and with greater awareness of their disease, or people with higher FOH (23). Even in such groups, higher scanning frequency is correlated with better glycemic outcomes.

We must acknowledge that our study has some limitations. First, the research was conducted in one center only and the sample size is small. Second, the study group was preselected as only adult T1DM patients paying for sensors out of pocket were included. Additionally, this group consisted of T1DM patients with good glycemic control who rarely experienced severe hypoglycemia within a year before the study entry. This group was characterized by an over-representation of female T1DM patients. This is related to the fact that they are attracted to our department by a special program dedicated to pregnancy planning and care. These women usually remain under our care after the delivery. However, in the study women currently planning pregnancy or being pregnant were not involved in the study. Moreover, no longitudinal data was analyzed, and no effect of previous sensors use, and patients’ experience was investigated. Next, due to the observational nature of our study, we cannot determine whether a cause-and-effect relationship exists between higher frequencies of daily scans and lower HFS II scores in adults with T1DM using isCGM. Such relationship could be established only in a future randomized clinical trial. However, the associations found in the current study are supported by previous reports on higher scanning frequencies and improvements in glycemic indices when using isCGM. Thus, patients who perform fewer daily scans could be advised to scan sensors more frequently to improve their glycemic control and reduce their FOH.

## Conclusion

For the first time, we report that higher scanning frequency is associated not only with improved glycemic indices but also with reduced FOH in adults with T1DM using isCGM. This constitutes a new argument for advising T1DM patients to undertake frequent scanning when using isCGM.

## Data availability statement

The raw data supporting the conclusions of this article will be made available by the authors, without undue reservation.

## Ethics statement

The studies involving human participants were reviewed and approved by The Bioethics Committee, The Medical Chamber in Krakow, Poland. The patients/participants provided their written informed consent to participate in this study.

## Author contributions

JH and MM designed the research. All authors were involved in acquisition of the data. JH and MM analysed the data and prepared the manuscript. All authors reviewed and accepted the final version of the manuscript and agreed to submit this version for publication. MM is the guarantor of the study.

## Acknowledgments

The authors would like to thank the whole team of the Department of Metabolic Diseases and Diabetology, University Hospital in Krakow that provide care for patients with diabetes, and all the patients who participated.

## Conflict of interest

KC, MM, PW, and TK have received fees from Abbott, Ascensia, Medtronic, Dexcom, Roche for lecturing and participating in the advisory panels. JH has received fees from Abbott, Ascensia, Dexcom, Roche for lecturing and participating in the advisory panels.

The remaining authors declare that the research was conducted in the absence of any commercial or financial relationships that could be construed as a potential conflict of interest.

## Publisher’s note

All claims expressed in this article are solely those of the authors and do not necessarily represent those of their affiliated organizations, or those of the publisher, the editors and the reviewers. Any product that may be evaluated in this article, or claim that may be made by its manufacturer, is not guaranteed or endorsed by the publisher.

## References

[1] Sun H Saeedi P Karuranga S Pinkepank M Ogurtsova K Duncan BB. IDF diabetes atlas: Global, regional and country-level diabetes prevalence estimates for 2021 and projections for 2045. Diabetes Res Clin Pract (2022) 183:109119. doi: 10.1016/j.diabres.2021.109119 34879977 PMC11057359

[2] Green A Hede SM Patterson CC Wild SH Imperatore G Roglic G. Type 1 diabetes in 2017: Global estimates of incident and prevalent cases in children and adults. Diabetologia (2021) 64(12):2741–50. doi: 10.1007/s00125-021-05571-8 PMC8563635 34599655

[3] Miller KM Beck RW Bergenstal RM Goland RS Haller MJ McGill JB. Evidence of a strong association between frequency of self-monitoring of blood glucose and hemoglobin A1c levels in T1D exchange clinic registry participants. Diabetes Care (2013) 36(7):2009–14. doi: 10.2337/dc12-1770 PMC3687326 23378621

[4] Schwandt A Best F Biester T Grünerbel A Kopp F Krakow D. Both the frequency of HbA1c testing and the frequency of self-monitoring of blood glucose predict metabolic control: A multicentre analysis of 15 199 adult type 1 diabetes patients from Germany and Austria. Diabetes Metab Res Rev (2017) 33(7):e2908. doi: 10.1002/dmrr.2908 28544457

[5] Araszkiewicz A Bandurska-Stankiewicz E Borys S Budzynski A Ogurtsova K Cyganek K 2022 guidelines on the management of diabetic patients. A position of diabetes Poland. Curr Top Diabetes (2022) 2(1):1–130.

[6] American Diabetes Association. 6. glycemic targets: Standards of medical care in diabetes — 2022. In: Diabetes care, vol. 45. (2022). p. S83–96. doi: 10.2337/dc22-S006

[7] FreeStyle libre system properties (Accessed 08.01.2021).

[8] Hohendorff J Gumprecht J Mysliwiec M Zozulinska-Ziolkiewicz D Malecki MT. Intermittently scanned continuous glucose monitoring data of polish patients from real-life conditions: More scanning and better glycemic control compared to worldwide data. Diabetes Technol Ther (2021) 23(8):577–85. doi: 10.1089/dia.2021.0034 PMC8377514 33794101

[9] Dunn TC Xu Y Hayter G Ajjan RA. Real-world flash glucose monitoring patterns and associations between self-monitoring frequency and glycaemic measures: A European analysis of over 60 million glucose tests. Diabetes Res Clin Pract (2018) 137:37–46. doi: 10.1016/j.diabres.2017.12.015 29278709

[10] Gomez-Peralta F Dunn T Landuyt K Xu Y Merino-Torres JF. Flash glucose monitoring reduces glycemic variability and hypoglycemia: Real-world data from Spain. BMJ Open Diabetes Res Care (2020) 8(1):e001052. doi: 10.1136/bmjdrc-2019-001052 PMC7103828 32198165

[11] Calliari LEP Krakauer M Vianna AGD Ram Y Barbieri DE Xu Y. Real-world flash glucose monitoring in Brazil: Can sensors make a difference in diabetes management in developing countries? Diabetol Metab Syndr (2020) 12:3. doi: 10.1186/s13098-019-0513-z 31921360 PMC6947827

[12] Battelino T Danne T Bergenstal RM Amiel SA Beck R Biester T. Clinical targets for continuous glucose monitoring data interpretation: Recommendations from the international consensus on time in range. Diabetes Care (2019) 42(8):1593–603. doi: 10.2337/dci19-0028 PMC6973648 31177185

[13] Bolinder J Antuna R Geelhoed-Duijvestijn P Kröger J Weitgasser R. Novel glucose-sensing technology and hypoglycaemia in type 1 diabetes: A multicentre, non-masked, randomised controlled trial. Lancet (2016) 388(10057):2254–63. doi: 10.1016/S0140-6736(16)31535-5 27634581

[14] Al Hayek AA Al Dawish MA. The potential impact of the FreeStyle libre flash glucose monitoring system on mental well-being and treatment satisfaction in patients with type 1 diabetes: A prospective study. Diabetes Ther (2019) 10(4):1239–48. doi: 10.1007/s13300-019-0616-4 PMC6612356 31066017

[15] Charleer S De Block C Van Huffel L Broos B Fieuws S Nobels F. Quality of life and glucose control after 1 year of nationwide reimbursement of intermittently scanned continuous glucose monitoring in adults living with type 1 diabetes (FUTURE): A prospective observational real-world cohort study. Diabetes Care (2020) 43(2):389–97. doi: 10.2337/dc19-1610 31843948

[16] Al Hayek AA Robert AA Al Dawish MA. Evaluation of FreeStyle libre flash glucose monitoring system on glycemic control, health-related quality of life, and fear of hypoglycemia in patients with type 1 diabetes. Clin Med Insights Endocrinol Diabetes. (2017) 10:1179551417746957. doi: 10.1177/1179551417746957 29270042 PMC5731614

[17] Martyn-Nemeth P Schwarz Farabi S Mihailescu D Nemeth J Quinn L. Fear of hypoglycemia in adults with type 1 diabetes: Impact of therapeutic advances and strategies for prevention - a review. J Diabetes Complications. (2016) 30(1):167–77. doi: 10.1016/j.jdiacomp.2015.09.003 26439754

[18] Böhme P Bertin E Cosson E Chevalier N GEODE group. Fear of hypoglycaemia in patients with type 1 diabetes: Do patients and diabetologists feel the same way? Diabetes Metab (2013) 39(1):63–70. doi: 10.1016/j.diabet.2012.10.006 23266467

[19] Martyn-Nemeth P Quinn L Penckofer S Park C Hofer V Burke L. Fear of hypoglycemia: Influence on glycemic variability and self-management behavior in young adults with type 1 diabetes. J Diabetes Complications (2017) 31(4):735–41. doi: 10.1016/j.jdiacomp.2016.12.015 PMC5350014 28143733

[20] Gonder-Frederick LA Schmidt KM Vajda KA Greear ML Singh H Shepard JA. Cox DJ psychometric properties of the hypoglycemia fear survey-ii for adults with type 1 diabetes. Diabetes Care (2011) 34(4):801–6. doi: 10.2337/dc10-1343 PMC3064031 21346182

[21] Rubin RR Peyrot M STAR 3 Study Group. Health-related quality of life and treatment satisfaction in the sensor-augmented pump therapy for A1C reduction 3 (STAR 3) trial. Diabetes Technol Ther (2012) 14(2):143–51. doi: 10.1089/dia.2011.0162 PMC4845679 22133037

[22] Hermanides J Nørgaard K Bruttomesso D Mathieu C Frid A Dayan CM. Sensor-augmented pump therapy lowers HbA(1c) in suboptimally controlled type 1 diabetes; A randomized controlled trial. Diabetes Med (2011) 28(10):1158–67. doi: 10.1111/j.1464-5491.2011.03256.x 21294770

[23] Rozporzadzenie ministra zdrowia z dnia 26 wrzesnia 2019 r. zmieniajace rozporzadzenie w sprawie wykazu wyrobow medycznych wydawanych na zlecenie. Dz.U. z 2019 r. poz. 1899 . Regulation of Minister of Health (Accessed 26.09.2020).

